# Early Buried Bumper Syndrome: A Rare Complication of Percutaneous Endoscopic Gastrostomy Tube Placement

**DOI:** 10.7759/cureus.9177

**Published:** 2020-07-14

**Authors:** Saeed Ali, Veysel Tahan, Ala Abdel Jalil

**Affiliations:** 1 Internal Medicine, University of Iowa, Iowa City, USA; 2 Gastroenterology, University of Missouri Columbia, Columbia, USA; 3 Gastroenterology and Hepatology, Creighton University, Omaha, USA

**Keywords:** dysphagia, buried bumper syndrome, peg tube placement, egd

## Abstract

Early buried bumper syndrome (BBS) is a rare complication of percutaneous endoscopic gastrostomy (PEG) tube placement where the internal bolster gets “buried” in the gastrocutaneous fistulous tract. BBS is usually a late complication with onset > four weeks of PEG placement. We present a case of early BBS presenting at day 17 after PEG tube placement where the internal bolster got embedded in the subcutaneous fat just outside the gastric wall. The patient underwent urgent endoscopic removal of the buried bumper with the simple external traction, followed by the successful placement of a new tube through the same tract. Early diagnosis and prompt management are of paramount importance to avoid an ominous outcome.

## Introduction

Percutaneous endoscopic gastrostomy (PEG) was first described by Guaderer and Ponsky in 1980 as an alternative to open surgery. It provides enteral access for patients who are not able to take food, water, or medications by mouth [1]. More than 250,000 PEG tubes are placed in the United States every year. Complications of PEG tube placement occur in 0.4%-22.5% of cases. It includes bleeding, infection, leakage, peritonitis, necrotizing fasciitis, tumor seeding, ileus, and buried bumper syndrome (BBS) [2].

BBS is a rare complication that occurs in 0.3%-2.4% of patients [3]. It was initially reported as a complication of PEG tube placement in 1988 [4,5]. It was later termed as “buried bumper” in 1990 [6]. This phenomenon occurs when the bolster inside the stomach causes ischemic necrosis of the gastric mucosa and migrates into the gastric wall or subcutaneous tissue [7].

BBS is a late complication of PEG tube placement with the majority of patients presenting after a year of the procedure. However, its presentation as early as eight days after the procedure has been reported. In our patient, it occurred approximately two weeks after the procedure [7-9].

## Case presentation

A 48-year-old woman with morbid obesity and persistent dysphagia after resection of a left jugular foramen schwannoma was referred for a PEG tube placement. The PEG tube was placed uneventfully with the external bolster placed at a 5-cm external mark. The patient presented to the emergency department (ED) four days later complaining of epigastric pain with minimal leakage around the PEG tube site. An x-ray abdomen with contrast showed the tube to be in a good position and flushing well. Three days later, the patient’s primary care physician prescribed clindamycin for the concern of PEG tube site infection.

**Figure 1:**
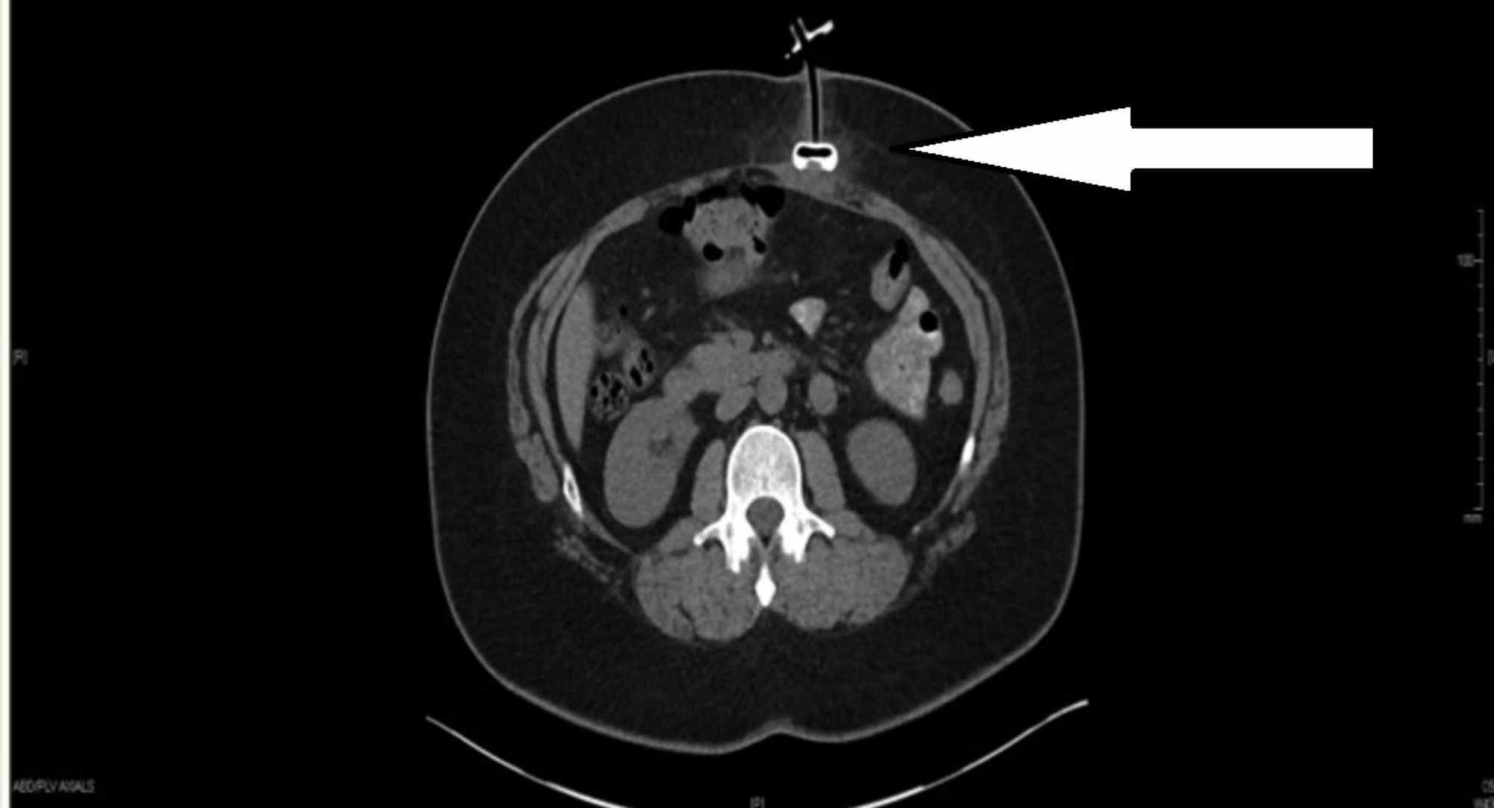
CT scan of the abdomen without contrast showing retraction of the internal bolster of the percutaneous endoscopic gastrostomy tube (solid white arrow) into the subcutaneous fat adjacent to the gastric wall.

After a week, the patient presented to the gastroenterology clinic complaining of continued leakage from the site around the tube. The external bolster was at a 6-cm external mark. The tube was re-inserted further with the external bolster tightened to a 4.5-cm external mark, and the patient was instructed to complete the course of clindamycin. The patient presented to the ED three days later with an inability to infuse tube feeding or even water. Physical examination showed normal vital signs. The abdomen was soft with no appreciable tenderness or clinical signs of peritonitis. There was no evidence of distention or palpable masses. There was no evidence of induration at the patient’s PEG site and the external bolster was noted at a 5.5-cm external mark with mild surrounding erythema. The patient underwent an abdominal x-ray with contrast which showed the tube to be outside the stomach with concern for retraction into the gastrocutaneous tract. Subsequently, a CT scan confirmed the retraction of the internal bolster into the subcutaneous fat just adjacent to the gastric wall (Figure 1).

**Figure 2:**
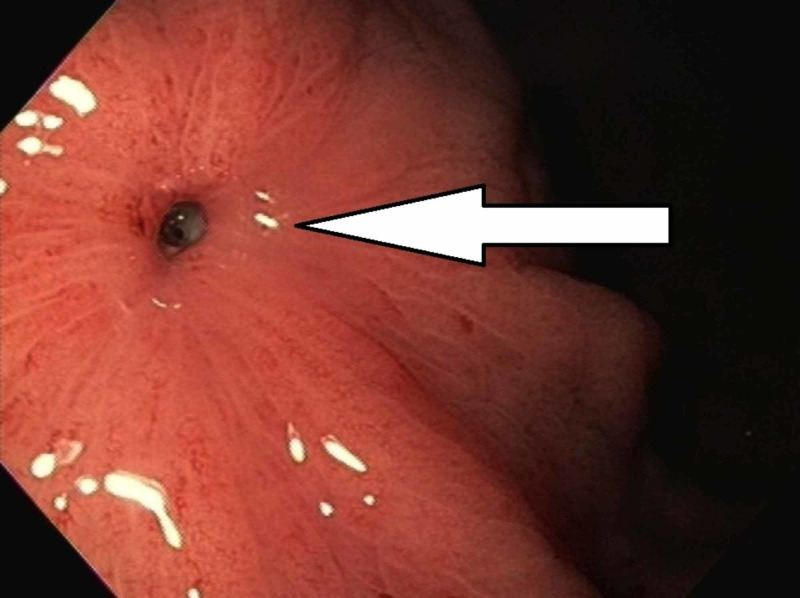
Endoscopic image showing percutaneous endoscopic gastrostomy tube site with a hole (solid white arrow) in the anterior stomach wall.

The patient was admitted to the hospital for intravenous (IV) antibiotics and fluid hydration. The patient underwent an esophagogastroduodenoscopy (EGD), which revealed a hole in the anterior wall of the gastric body (Figure 2).

**Figure 3:**
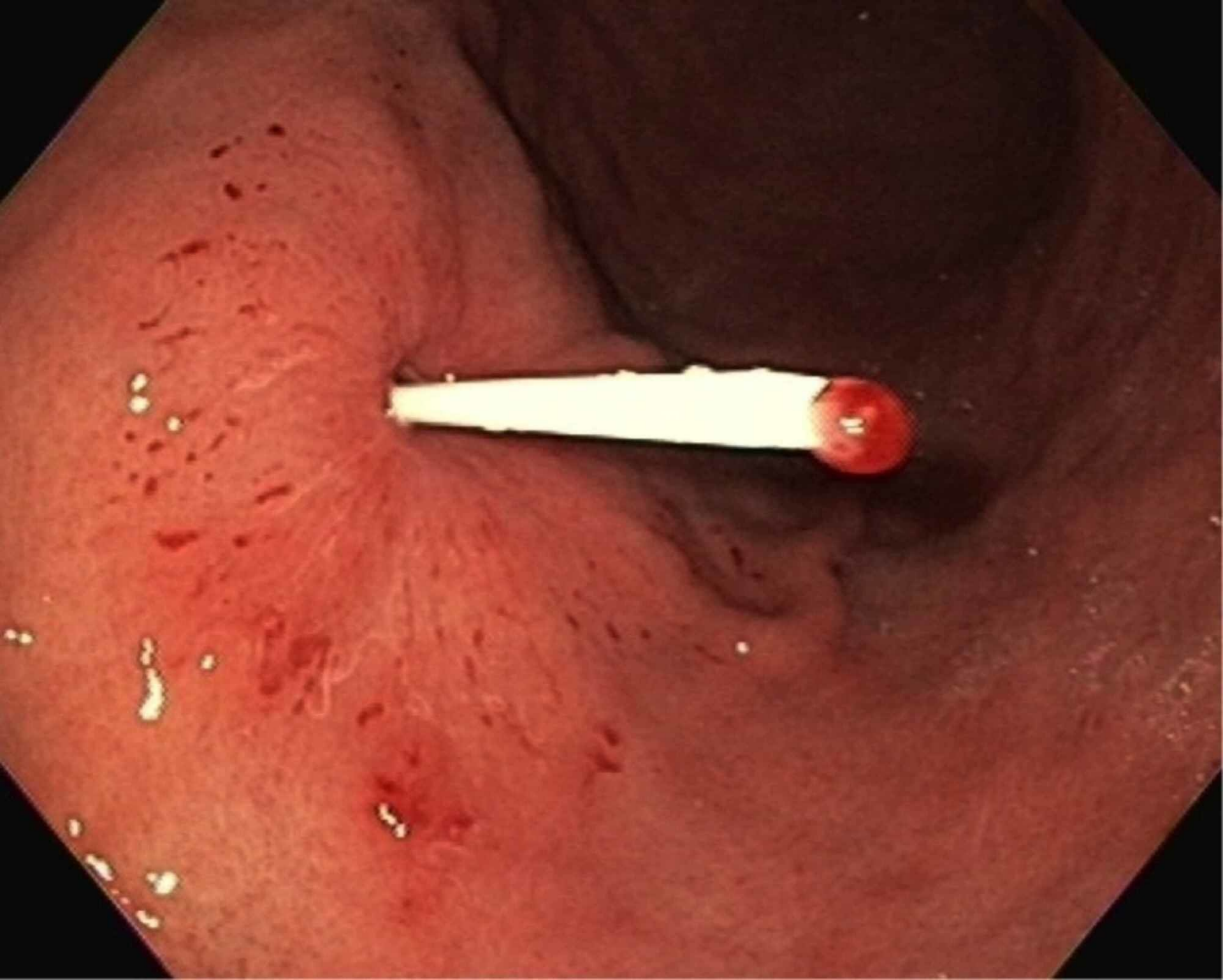
Endoscopic image showing a trocar that was introduced through the original gastrostomy orifice to place a new percutaneous endoscopic gastrostomy tube.

The PEG tube was removed externally with simple traction. A plastic trocar (without needle) was used to check the integrity of the tract (Figure 3).

A guidewire was introduced into the stomach and snared, and a PEG tube was placed through the same tract with the external bolster left at a 4-cm external mark. Tube feeding was initiated 24 hours later, with recommendations not to remove the PEG tube for 12 weeks, and to complete the course of the antibiotics. The patient was discharged home in two days. At a follow-up visit several weeks later, she was tolerating tube feedings well without any complication.

## Discussion

BBS represents a less common but a major complication of the PEG tube placement. To date around 12 cases of early BBS (≤ four weeks) have been described in the literature with onset as early as eight days after tube placement [7]. Traction on the internal bolster during placement, manipulation, or by the abdominal adipose tissue when it sags in the upright posture appears to be the etiology of this syndrome. This traction creates pressure between the internal bolster and the gastric wall, causing pressure necrosis. Other risk factors include obesity, multiple gauzes or other dressings between the external bolster and the abdominal wall, manipulation of the tube by inexperienced personnel, and even chronic cough. Traction on the tube caused by inadvertent tugging or extreme tightness of the external bolster has been attributed as a major cause [5,6,10,11]. PEG tube design was found not to account for BBS, but other evidence suggested that the design of certain PEG tubes (a rigid crossbar, stiff flaps, or a small bolster) may pose a risk for BBS [7,12].

The classic symptoms constellation of BBS includes tube blockage, peritubal leakage, resistance to infuse feedings, and abdominal pain [10]. It can also present as an abscess, abdominal wall infection, peritonitis, gastrointestinal bleeding, or sepsis. There has been a case report of death resulting from the syndrome [13].

Its presentation overlaps with the other PEG tube complications; however, a careful physical exam can differentiate BBS from other complications. Buried bumper causes the tube to become fixed in place; therefore, on examination, the PEG tube cannot rotate within or slide through the stoma, and hence it is mainly a clinical diagnosis [14]. In contrast, PEG tube is freely movable on examination in patients with localized wound infection, uncomplicated PEG tube leakage, and tube obstruction, and is not painful with the instillation of fluids. Imaging modalities like CT scan and tube studies aid the diagnosis of BBS and can confirm the exact location of the tube [15].

The definitive treatment of BBS is the removal of the PEG tube. Several techniques have been described for the management of BBS, which include endoscopic methods, surgical techniques, or combination of the two [8,12,13]. The location and depth of the buried bumper, clinical presentation, and patient’s comorbidities guide the decision for management [16].

Preventive measures include adequate positioning of the external bolster, allowing a distance of at least 10 mm between the skin and the external bolster to minimize the risk of pressure necrosis [2]. “PEG twirl sign” is another important preventive measure that can be used once the stoma channel is matured (usually in two weeks). Here, the external bolster is unfastened once weekly, PEG tube is inserted several centimeters inside, and turned 360 degrees around its long axis, and the external bolster is fastened back to the original loose position. This maneuver is however not suitable for balloon catheters and PEG with a jejunal extension [17]. Similarly, better communication among the involved subjects, such as patients, relatives, nurses, home health staff, nursing home staff, endoscopists, and dieticians, is the key to prevention [18].

The abstract of this article had been presented at the World Congress of Gastroenterology at American College of Gastroenterology (ACG) meeting​ in Orlando, Florida in October 2017 [19].

## Conclusions

Early identification and management of BBS is crucial to prevent further complications. Our case describes successful management of early BBS for a patient who had a lingering course with the feeding tube since the time of its original placement where continuous traction and manipulation resulted in the retraction of the tube’s inner bumper into the outer gastric wall/subcutaneous fat. We used simple external traction to remove the buried bumper without any complications. The majority of published cases of early BBS required more aggressive endoscopic or surgical interventions. The prompt recognition and treatment of BBS help avoid more invasive endoscopic or surgical measures. 
